# Supplementary material for: Menstrual hygiene management practices after the chhaugoth demolition campaign in Achham, Nepal
Source: PLoS One. 2026 Jun 3;21(6):e0336461. doi: 10.1371/journal.pone.0336461 (PMC13232791; doi:10.1371/journal.pone.0336461)
Supplement: S1 — (PDF) [file pone.0336461.s001.pdf]

Achham Project

वडा नंः

____

तपाईले छाउपडी प्रथाको बारेमा सुन्नुभएको छ ?

- ◯ छ
- ◯ छैन्

के तपाइँ कहिल्यै छाउगोठमा बस्नुभएको छ?

- ◯ छ
- ◯ छैन्

छाउगोठको अर्थ थाहा नभएको भए पनि छाउगोठमा बसेको भए अन्तर्वार्ताको लागी अगाडी बढ्नुहोस्

A.सामाजिक जनसांख्यिक विवरण

---

१. उमेर (पुरा भएको वर्ष):

____

२. जातियता

- ◯ अन्य (खुलाउनु होस्)
- ◯ दलित
- ◯ ब्राहमण
- ◯ जनजाती
- ◯ क्षेत्री

३. धर्म:

- ◯ हिन्दु
- ◯ इस्लाम/मुस्लिम
- ◯ बौद्ध
- ◯ क्रिशियन
- ◯ अन्य (खुलाउनु होस्)

४. वैवाहिक स्थिति:

- ◯ अबिबाहित
- ◯ विवाहित
- ◯ सम्बन्धविच्छेद (विछोड)
- ◯ विधवा

५. परिवारका किसिम:

- ◯ सयुक्त परिवार
- ◯ एकल परिवार

६.शैक्षिक स्थिति:

- ◯ निरक्षर
- ◯ अनौपचारिक
- ◯ आधारभूत
- ◯ माध्यमिक
- ◯ स्नातक
- ◯ स्नातकोत्तर वा सो भन्दा माथि

७.पेशा (अन्तर्वार्ता दिने व्यक्तिको):

- ◯ गृहिणी
- ◯ बिद्यार्थी
- ◯ व्यावसायिक
- ◯ किसान
- ◯ अन्य

८.घरको कुल मासिक आय (NRs):

____

९.बुबा शैक्षिक स्थिति:

- ◯ निरक्षर
- ◯ अनौपचारिक
- ◯ आधारभूत
- ◯ माध्यमिक
- ◯ स्नातक
- ◯ स्नातकोत्तर वा सो भन्दा माथि

१०.ससुराको शैक्षिक स्थिति:

- निरक्षर
- अनौपचारिक
- आधारभूत
- माध्यमिक
- स्नातक
- स्नातकोत्तर वा सो भन्दा माथि

११.आमाको शैक्षिक स्थिति:

- निरक्षर
- अनौपचारिक
- आधारभूत
- माध्यमिक
- स्नातक
- स्नातकोत्तर वा सो भन्दा माथि

१२.सासुको शैक्षिक स्थिति:

- निरक्षर
- अनौपचारिक
- आधारभूत
- माध्यमिक
- स्नातक
- स्नातकोत्तर वा सो भन्दा माथि

१३.वुवाको पेसा:

- कृषि
- व्यवसाय
- सेवा
- बैदेशिक रोजगार
- दैनिक मजदुरि
- सेवानिवृत्त/रिटायर्ड/पेन्सन
- बेरोजगार
- अन्य

१४.ससुराको पेसा:

- ◯ कृषि
- ◯ व्यवसाय
- ◯ सेवा
- ◯ बैदेशिक रोजगार
- ◯ दैनिक मजदुरि
- ◯ सेवानिवृत्त/रिटायर्ड/पेन्सन
- ◯ बेरोजगार
- ◯ अन्य

१५.आमाको पेसा:

- ◯ कृषि
- ◯ व्यवसाय
- ◯ सेवा
- ◯ बैदेशिक रोजगार
- ◯ दैनिक मजदुरि
- ◯ सेवानिवृत्त/रिटायर्ड/पेन्सन
- ◯ बेरोजगार
- ◯ अन्य

१६.सासुको पेसा:

- ◯ कृषि
- ◯ व्यवसाय
- ◯ सेवा
- ◯ बैदेशिक रोजगार
- ◯ दैनिक मजदुरि
- ◯ सेवानिवृत्त/रिटायर्ड/पेन्सन
- ◯ बेरोजगार
- ◯ अन्य

१७.पतिको शौक्षिक स्थिति (विवाहितका लागि मात्र)

- निरक्षर
- अनौपचारिक
- आधारभूत
- माध्यमिक
- स्नातक
- स्नातकोत्तर वा सो भन्दा माथी

१८.पतिको पेशा (विवाहितका लागि मात्र):

- कृषि
- व्यवसाय
- सेवा
- बैदेशिक रोजगार
- दैनिक मजदुरि
- सेवानिवृत्त
- बेरोजगार
- अन्य

B.बस्ने व्यवस्था सम्बन्धी जानकारी

---

१. छाउगोठ उन्मूलन हुनु अघि तपाई महिलाबारि हुँदा कहाँ बस्नुभएको थियो ?

- छाउगोठ
- एउटै घरमा छुट्टै कोठा
- अन्य (Specify)

१ क. यदि छाउगोठमा बस्नुभयो भने, महिनावारीको समयमा छाउगोठमा बसेको औसत समय कति थियो?

- ४ दिन
- ५ दिन
- ६ दिन
- अन्य (Specify)

२. सुरक्षाको दृष्टिकोणबाट हेर्दा महिनावारीको अवधिमा छाउगोठमा बस्नु कस्तो हो?

- सुरक्षित
- असुरक्षित
- अन्य (Specify)

३. छाउगोठ उन्मूलन हुनु अघि महिनावारीको समयमा सुत्नको लागि तपाईले कस्तो प्रकारको लुगा/सामग्री प्रयोग गर्नुभयो?

- ○ न्यानो लुगाहरु
- ○ पराल
- ○ स्थानीय गद्दा (Motto/matrices)
- ○ खाली भुइँ
- ○ अन्य (Specify)

४. छाउगोठको उन्मूलन अभियान पछि तपाई कहाँ बस्नुहुन्छ?

- ○ छाउगोठ
- ○ घरमै छुट्टै कोठा
- ○ अन्य (निर्दिष्ट)

४ क.यदि छाउगोठमा बस्नुभयो भने, छाउगोठ उन्मूलन भएपछि महिनावारीको समयमा छाउगोठमा बसेको औसत समय कति थियो ?

- ○ ४ दिन
- ○ ५ दिन
- ○ ६ दिन
- ○ अन्य (निर्दिष्ट)

४ ख. यदि छाउगोठमा बस्नुभयो भने, तपाइँ आफ्नो महिनावारी चक्रमा कति पटक त्यहाँ बस्नुहुन्छ?

____

४ ग. यदि तपाईं अन्य ठाउँमा बस्नुहुन्छ भने, कृपया महिनावारीको समयमा बस्नको लागि हालको ठाउँ उल्लेख गर्नुहोस्?

____

४ घ. महिनावारीको समयमा हालको बस्ने ठाउँमा सुरक्षाको बारेमा तपाई कस्तो महसुस गर्नुहुन्छ?

- ○ सुरक्षित
- ○ असुरक्षित
- ○ अन्य (Specify)

५. छाउगोठ भत्काए पछि महिनावारीको समयमा सुत्नको लागि तपाईले कस्ता कपडा/सामग्री प्रयोग गर्नुभयो?

- ☐ न्यानो लुगाहरू
- ☐ पराल
- ☐ स्थानीय गद्दा
- ☐ खाली भुई
- ☐ अन्य (specify)

५. छाउगोठ भत्काए पछि महिनावारीको समयमा सुत्नको लागि तपाईले कस्ता कपडा/सामग्री प्रयोग गर्नुभयो?

- ◯ न्यानो लुगाहरु
- ◯ पराल
- ◯ स्थानीय गद्दा (मोटो)
- ◯ खाली भुइँ
- ◯ अन्य (निर्दिष्ट)

६. छाउपडी प्रथाको उन्मूलनअभियान पछि तपाईंले आफ्नो महिनावारीको समयमा पालना गर्नुपर्ने कुनै प्रवधानहरू छन् ।

- ◯ छन
- ◯ छैन्न

६ क. यदि हो भने ती प्रवधानहरू/परम्पराहरू के हुन्?

____

७ क. महिनावारीको समयमा आफ्नो हालको जीवनयापनलाई कसरी मूल्याङ्कन गर्नुहुन्छ?

- ◯ जबरजस्ती अपनायो
- ◯ परम्पराको रूपमा स्वीकार गरिएको छ
- ◯ भाग्यको रूपमा स्वीकार गरियो
- ◯ अन्य

८.छाउगोठ भत्काउने सरकारको पहलले तपाईको छाउगोठ पनि भत्कायो ?

- ◯ भत्कायो
- ◯ भत्कायेन

C. महिनावारी स्वच्छता व्यवस्थापन अभ्यास

____

१.महिनावारीको राकता रक्तश्राव व्यवस्थापन गर्नको लागि तपाइँ के प्रयोग गर्नुहुन्छ कुन महिनावारी स्वच्छता Products प्रयोग गर्नुहुन्छ

- ☐ स्थानीय रूपमा तयार कपडा प्याड
- ☐ पुरानो लुगा (टालो)
- ☐ सेनेटरी प्याड
- ☐ महिनावारी कपहरू
- ☐ प्रयोग गर्न केहि छैन
- ☐ अन्य (Specify)

१ क. यदि माथि दिइका वस्तुहरु(a,c,d) प्रयोग गरिन्छ, भने उक्त बस्तुको पहुच कस्तो रहेको छ ?

- ◯ सजिलै पहुँचयोग्य
- ◯ मुश्किलले पहुँचयोग्य
- ◯ पहुँचयोग्य छैन
- ◯ अन्य

२. के तपाईंले सुरक्षित महिनावारी स्वच्छता अभ्यासहरू बारे कुनै शिक्षा, जानकारी वा तालिम प्राप्त गर्नुभएको छ?

- ◯ छ
- ◯ छैन

३. के तपाईंले छाउगोठ भत्काउने अभियान पछि आफ्नो समुदायमा महिनावारी सरसफाइ अभ्यासमा कुनै परिवर्तन देख्नुभएको छ?

- ◯ छ
- ◯ छैन

३क. यदि छ भने, तपाईंले कस्तो प्रकारका परिवर्तनहरू अवलोकन गर्नुभएको छ? उल्लेख गर्नुहोस्

____

४. के तपाईं छाउगोठ महिनावारी उन्मूलन अभियान पछि कहिल्यै महिनावारि हुदाँ छाउगोठमा बस्नुभएको छ?

- ◯ छ
- ◯ छैन

५. तपाइँले आफ्नो महिनावारी चक्र को समयमा सेनेटरी Products कति पटक परिवर्तन गर्नुहुन्छ?

- ◯ दिनमा एक पटक
- ◯ दिनमा दुई पटक
- ◯ दिनमा तीन पटक
- ◯ दिनमा तीन पटकभन्दा बढी
- ◯ आवश्यक अनुसार
- ◯ अन्य

६. के तपाइँसँग महिनावारीको समयमा सरसफाइका लागि सफा पानी र साबुनको पहुँच कस्तो रहेको छ ?

- ◯ सधैं पहुँचयोग्य
- ◯ कहिलेकाहीं पहुँचयोग्य
- ◯ पहुँचयोग्य छैन
- ◯ अन्य

७. महिनावारीको समयमा उत्पादन हुने प्रयोग गरिएका सामग्री/फोहोरलाई तपाईं कहाँ फाल्नुहुन्छ?

____

८. के तपाईलाई हालको डिस्पोजल अभ्यास सुरक्षित छ जस्तो लाग्छ?

- ◯ सुरक्षित छ
- ◯ सुरक्षित छैन
- ◯ थाहा छैन
- ◯ अन्य

९. के तपाइँ आफ्नो महिनावारी सम्बन्धी समस्याहरू अरूसँग छलफल/साझेदारी गर्नुहुन्छ?

- ◯ गर्छु
- ◯ गर्दिन

९ क. यदि हो भने, तपाईं कोसँग छलफल गर्नुहुन्छ?

- ◯ साथी समूह
- ◯ परिवारका सदस्यहरु
- ◯ शिक्षक/शिक्षिका
- ◯ स्वास्थ्य कर्मि
- ◯ अन्य (Specify)

१०. के तपाईंको समुदायमा महिलाहरूलाई महिनावारि स्वच्छता व्यवस्थापन गर्न प्रतिबन्धित गर्ने कुनै सांस्कृतिक/सामाजिक विश्वासहरू छन्?

- ◯ छन
- ◯ छैन्न

१० क.यदि छन् भने खुलाउनु होस।

____

११. के तपाइँ महिनावारीको समयमा नुहाउनुहुन्छ?

- ◯ नुहाउछु
- ◯ नुहाउदिन

११ क. यदि हो भने, महिनावारी भएको वेला कति दिन नुहाउनुहुन्छ?

- ◯ पहिलो दिन
- ◯ दोस्रो दिन
- ◯ तेस्रो दिन
- ◯ चौथो दिन
- ◯ अन्य

११ ख.यदि हो भने महिनावारी भएको बेला पहिलो पटक नुहाएर कति दिनसम्म नुहाउनु हुन्छ ?

- एक दिनको लागि
- दुई दिन नियमित
- तीन दिनको लागि नियमित
- अन्य (निर्दिष्ट)

१२. महिनावारीको समयमा तपाई कहाँ दिसा र पिसाब गर्नुहुन्छ?

- घरको साझा शौचालय
- घरको छुट्टै शौचालय
- खुल्ला ठाउँ
- अन्य (निर्दिष्ट)

१३ के तपाइँ महिनावारीको अवधिमा गोप्यअंग/गुप्ताङ्ग सफा गर्नुहुन्छ?

- गर्छु
- गर्दिन

१३ क. यदि गर्नुहुन्छ भने, KOमहिनावारीको अवधिमा एक दिनमा कति पटक आफ्नो गोप्यअंग/गुप्ताङ्ग सफा गर्नुहुन्छ?

- दिनमा एकपटक
- दिनको दुई पटक
- दुई भन्दा बढि

१४. महिनावारीको समयमा यौनाङ्ग सफा गर्न कुन सामाग्रि प्रयोग गर्नुहुन्छ?

- साबुन र पानी
- पानी मात्र
- सादा कागज
- अन्य

१५. छाउपडी प्रथा अन्त्यका लागि छाउगोठ भत्काउने अभियानको कार्यान्वयन अवस्थालाई कसरी मूल्याङ्कन गर्नुहुन्छ ?

- पूर्ण रूपमा कार्यान्वयन (छाउपडी प्रथा समाप्त भयो)
- आंशिक रूपमा लागू (कतिपय घरायसी छाउपडी चलन हट्यो)
- कुनै परिवर्तन अवलोकन गरिएको छैन
- अन्य

D.. छाउपडि बारे धारणा

१.छाउपडी अभ्यास गर्न महिलाहरूलाई स्वीकार गरिन्छ

- ◯ पूर्ण सहमत
- ◯ सहमत
- ◯ असहमत
- ◯ पूर्ण असहमत
- ◯ न सहमत न असहमत

२.महिनावारी हुँदा महिलाले छुट्टाछुट्टै खानेकुरा खानुपर्छ

- ◯ पूर्ण सहमत
- ◯ सहमत
- ◯ असहमत
- ◯ पूर्ण असहमत
- ◯ न सहमत न असहमत

३.महिनावारी हुँदा महिलाले दुग्धजन्य पदार्थ खानु हुदैन् ।

- ◯ पूर्ण सहमत
- ◯ सहमत
- ◯ पूर्ण असहमत
- ◯ असहमत
- ◯ न सहमत न असहमत

४.महिनावारीको चौथो दिनमा प्रयोग गरिसकेपछि सबै ओछ्यानका लुगाहरू सफा गर्न महत्त्वपूर्ण छ

- ◯ पूर्ण सहमत
- ◯ सहमत
- ◯ पूर्ण असहमत
- ◯ असहमत
- ◯ न सहमत न असहमत

५.महिनावारीको समयमा लगाउनको लागि विशेष कपडाहरू राख्नु पर्छ

- ◯ पूर्ण सहमत
- ◯ सहमत
- ◯ पूर्ण असहमत
- ◯ असहमत
- ◯ न सहमत न असहमत

६.महिनावारीको बेला महिलाहरू भान्साकोठामा जानु हुदैन् ।

- ◯ पूर्ण सहमत
- ◯ सहमत
- ◯ पूर्ण असहमत
- ◯ असहमत
- ◯ न सहमत न असहमत

७.महिनावारी भएको बेला महिलाहरु पूजा गर्न जानु हुँदैन

- ◯ पूर्ण सहमत
- ◯ सहमत
- ◯ पूर्ण असहमत
- ◯ असहमत
- ◯ न सहमत न असहमत

८.महिनावारी भएको बेला धार्मिक सभामा जानु हुदैन् ।

- ◯ पूर्ण सहमत
- ◯ सहमत
- ◯ पूर्ण असहमत
- ◯ असहमत
- ◯ न सहमत न असहमत

९.महिलाले महिनावारीको अवस्था भएपनि आफूले चाहेको ठाउँमा जाना पाउनुपर्छ ।

- ◯ पूर्ण सहमत
- ◯ सहमत
- ◯ पूर्ण असहमत
- ◯ असहमत
- ◯ न सहमत न असहमत

१०.महिनावारी हुँदा महिलाहरूले घरको साझा शौचालय प्रयोग गर्नुपर्छ ।

- ◯ पूर्ण सहमत
- ◯ सहमत
- ◯ पूर्ण असहमत
- ◯ असहमत
- ◯ न सहमत न असहमत

११.महिनावारी हुँदा महिलाहरूलाई भान्साकोठामा खाना पकाउन नजान नदिनु उहाहरुलाई मासिक विश्राम हो ।

- पूर्ण सहमत
- सहमत
- पूर्ण असहमत
- असहमत
- न सहमत न असहमत

१२.महिनावारी हुँदा महिलाले खाना पकाउनु नपर्दा उहाहरुलाई राम्रो हुन्छ

- पूर्ण सहमत
- सहमत
- पूर्ण असहमत
- असहमत
- न सहमत न असहमत

१३.महिनावारि भएको बेला महिलाले प्रशास्त आराम गर्नुपर्छ ।

- पूर्ण सहमत
- सहमत
- पूर्ण असहमत
- असहमत
- न सहमत न असहमत

E. Observation Checklist

१.बस्ने ठाउँहरूमा भेन्टिलेसन/झ्यालहरूको उपलब्धता:

- छ
- छैन्

२.ढोका र झ्यालहरूमा लकको उपलब्धता::

- छ
- छैन्

३.प्रयोग गरिएको प्याड शोषक डिस्पोज गर्ने ठाउँ:

- छ
- छैन्

४.बस्ने ठाउँहरूमा सुत्ने व्यवस्था:

- ◯ छ
- ◯ छैन्

५.बस्ने ठाउँहरूमा सामान्य रूपमा खानाको उपलब्धता:

- ◯ छ
- ◯ छैन्

६.बस्ने ठाउँहरूमा सामान्य रूपमा पिउने पानीको उपलब्धता:

- ◯ छ
- ◯ छैन्

७.बस्ने ठाउँ भित्र प्रकाशको व्यवस्था:

- ◯ छ
- ◯ छैन्

८.बस्ने ठाउँहरूमा बिजुलीको उपलब्धता:

- ◯ छ
- ◯ छैन्

९.बस्ने ठाउँमा शौचालयको व्यवस्था:

- ◯ छ
- ◯ छैन्

९क.यदि हो भने, यो महिनावारी अवधिमा प्रयोग गर्नको लागि अलग छ:

- ◯ छ
- ◯ छैन्

१०. बस्ने ठाउँबाट शौचालयको दूरी:

____

११. नजिकैको घर/समुदायबाट बस्ने ठाउँको दूरी:

- ◯ ५० मि भन्दा कम
- ◯ ५०-१०० मिटर
- ◯ १०० मिटर भन्दा धैरै

१२.बस्ने ठाउँहरूमा, महिनावारीको रगतको प्रवाह रोक्न प्रयोग हुने लुगा सुकाउनको लागि ठाउँको उपलब्धता:

- ◯ छ
- ◯ छैन्
