# Supplementary material for: Menstrual hygiene management practices after the chhaugoth demolition campaign in Achham, Nepal
Source: PLoS One. 2026 Jun 3;21(6):e0336461. doi: 10.1371/journal.pone.0336461 (PMC13232791; doi:10.1371/journal.pone.0336461)
Supplement: Attachment — Submitted filename: Reviewer Comments on Manuscript.pdf [file pone.0336461.s004.pdf]

Reviewer Comments on Manuscript

Thank you for the opportunity to review this timely manuscript examining menstrual hygiene management (MHM) practices in Achham, Nepal after the chhaugoth demolition campaign. The authors are to be commended for engaging with a culturally rich and complex topic with significant implications for adolescent wellbeing and community health. Empirical evidence emerging from interventions, such as chhaugoth demolition campaigns, is especially valuable for advancing the field, and the focus on post-intervention practices has the potential to meaningfully inform future programs and policy efforts.

Below, I provide a series of comments and suggestions aimed at strengthening the clarity, methodological rigor, and contextual depth of the manuscript. Overall, there are several key components of the paper that I would suggest significantly expanding/reworking to be ready for publication, and hence suggest a major revision.

Abstract

- Please exercise caution in how the term *chhaupadi* is introduced in the opening sentence. Chhaupadi is a complex, locally situated practice, and this complexity requires greater clarity. The manuscript introduction does a better job of defining chhaupadi; I recommend ensuring consistency between the abstract and introduction.
- Grammar and word choice throughout the abstract requires improvement for readability (e.g., “extricable” should be “inextricably,” and many additional other examples throughout).
- Please specify the year(s) in which the chhaugoth demolition campaign was implemented and clarify how this timing relates to the period of data collection.
- Clarify participant sampling by specifying whether participants were selected from all wards. Consider replacing “different wards” with a more explicit description.
- Grammar throughout the Results portion of the abstract needs substantial revision, as it is currently difficult to follow.

Introduction

- Writing and grammar throughout the manuscript need careful revision to improve clarity and flow.
- It is not sufficiently clear what the chhaugoth demolition campaign entailed. Please provide additional detail on the intervention, including:
    - Who implemented the campaign
    - What specific activities were included
    - The duration and geographic scope of the campaign
    - The extent of community engagement

    Including this information earlier in the introduction would help readers better interpret the results.
- Lines 109–112, which describe the broader health implications of chhaupadi, may be better positioned earlier in the introduction to help frame the public health relevance of the study.
- Critically, the introduction does not adequately address the complexities and unintended consequences of shed demolition and the chhaugoth campaign. Prior research has documented that some women report having *no alternative shelter* following demolition. This potential harm—and community concerns around it—should be acknowledged upfront to provide a more balanced framing of the intervention.

Methods

- Line 121 refers to “subsequent events”; please clarify what these events were.
- The authors note that the study area was selected due to the reported prevalence of chhaupadi and inclusion in government demolition campaigns. However, other municipalities likely met these criteria. Please explain how this specific municipality was selected (e.g., government recommendation, logistical considerations).
- The assumed chhaupadi prevalence of 50% used for sample size estimation requires citation.
- Please describe enumerator training in greater detail, including duration, content, and any steps taken to ensure data quality. Additionally, provide information on enumerators’ gender and other relevant positionality characteristics.
- Lines 144–146 describe data collected on MHM practices. Given that chhaupadi practices are also a central focus of the paper, please clarify what

questions were asked regarding isolation practices (e.g., sleeping location, restrictions, duration) and consider providing example questions.

- Please review and revise language related to consent procedures for participants under 18 years of age; typically, both assent and parental/guardian consent are required.
- The current analysis relies primarily on descriptive statistics, which provides a useful overview of participant characteristics and MHM practices. However, the analytic approach could be strengthened by incorporating inferential statistical tests to examine associations between participant characteristics and key behaviors.
- Specifically, the authors may consider conducting bivariate analyses to assess whether sociodemographic factors (e.g., age, education, marital status, household income, or location) are associated with outcomes such as menstrual product use, frequency of changing materials, hygiene and disposal practices, or sleeping in a chhaugoth. Chi-square tests could be used for categorical variables, with t-tests, ANOVA, or appropriate nonparametric alternatives for continuous variables.
- Where sample size and data structure allow, multivariable analyses (e.g., logistic regression) could further strengthen the findings by identifying independent predictors of specific MHM or chhaupadi-related behaviors while controlling for potential confounders.
- If inferential analyses are not feasible, the authors should explicitly justify this limitation and clarify the exploratory nature of the analysis from the outset.

Results

- In Table 1, the presentation of monthly household income is difficult to interpret and does not follow the formatting used for other sociodemographic variables. I recommend revising this for consistency and clarity.
- The results suggest a substantial reduction in the number of women and girls sleeping in chhaugoths. Given that chhaupadi is criminalized, please describe what steps were taken to minimize social desirability bias during data collection. Additional discussion is needed to support confidence in these findings.
- In Table 2, the definition of "practices or restrictions" is unclear. It is difficult to reconcile findings suggesting no current restrictions with existing literature, particularly if religious restrictions (e.g., avoiding temples or religious activities)

were included. Please clarify how this question was framed and explained to participants so that readers can accurately interpret the results.

References and Contextual Literature

Several key bodies of literature central to understanding chhaupadi and its complexities are missing. The manuscript would be significantly strengthened by incorporating relevant foundational and contemporary sources, including:

- Seminal work on purity and pollution, such as:
  - Cameron, M. *On the Edge of Auspicious: Gender and Caste in Nepal*.
- Relevant Nepali scholarship on menstrual restriction and resistance, for example:
  - https://nepjol.info/index.php/ajps/article/view/51094
- Research by Baumann et al. addressing multiple dimensions of chhaupadi that are directly relevant to this manuscript, including:
  - Criminalization and policy implications
    - https://pubmed.ncbi.nlm.nih.gov/34051090/
    - https://blogs.lshtm.ac.uk/hppdebated/2021/06/01/is-criminalization-the-answer-for-ending-a-harmful-practice-in-nepal/
  - Cultural dimensions of chhaupadi
    - https://www.tandfonline.com/doi/full/10.1080/23293691.2020.1861413
  - Intervention development and community-engaged approaches
    - https://doi.org/10.1080/13691058.2024.2373793
- Budha's work, which offers important contextual insight into chhaupadi practices:
  - https://www.dbpia.co.kr/journal/articleDetail?nodeId=NODE07365082
- I also recommend consulting and citing the recent comprehensive review by Joshi et al.:
  - https://onlinelibrary.wiley.com/doi/epdf/10.1002/wmh3.491
